# Early stage fatigue damage occurs in bovine tendon fascicles in the absence of changes in mechanics at either the gross or micro-structural level

**DOI:** 10.1016/j.jmbbm.2014.06.005

**Published:** 2014-10

**Authors:** Jennifer H. Shepherd, Graham P. Riley, Hazel R.C. Screen

**Affiliations:** a Institute of Bioengineering, School of Engineering and Materials Science, Queen Mary, University of London, Mile End Road, E1 4NS, UK; b School of Biological Sciences, University of East Anglia, UK

## Abstract

Many tendon injuries are believed to result from repetitive motion or overuse, leading to the accumulation of micro-damage over time. *In vitro* fatigue loading can be used to characterise damage during repeated use and investigate how this may relate to the aetiology of tendinopathy.

This study considered the effect of fatigue loading on fascicles from two functionally distinct bovine tendons: the digital extensor and deep digital flexor. Micro-scale extension mechanisms were investigated in fascicles before or after a period of cyclic creep loading, comparing two different measurement techniques – the displacement of a photo-bleached grid and the use of nuclei as fiducial markers.

Whilst visual damage was clearly identified after only 300 cycles of creep loading, these visual changes did not affect either gross fascicle mechanics or fascicle microstructural extension mechanisms over the 900 fatigue cycles investigated. However, significantly greater fibre sliding was measured when observing grid deformation rather than the analysis of nuclei movement. Measurement of microstructural extension with both techniques was localised and this may explain the absence of change in microstructural deformation in response to fatigue loading. Alternatively, the data may demonstrate that fascicles can withstand a degree of matrix disruption with no impact on mechanics. Whilst use of a photo-bleached grid to directly measure the collagen is the best indicator of matrix deformation, nuclei tracking may provide a better measure of the strain perceived directly by the cells.

## Highlights

• Tendon fascicle gross mechanics and micro-scale deformation investigated after fatigue loading.
• Fascicles can withstand a degree of matrix disruption without impact on mechanics.
• More fibre sliding was observed measuring grid deformation than tracking nuclei.
• Nuclei tracking may better represent the strains experienced by cells than grid deformation.

## Graphical abstract

**Figure:**
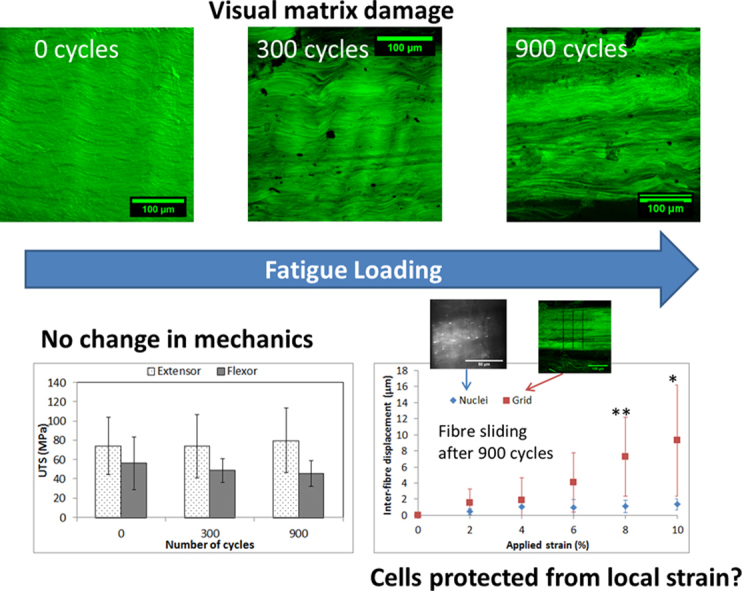

## 1 Introduction

Many tendon injuries (tendinopathies) are believed to result from repetitive motion, or overuse, which creates ‘micro-trauma’ that accumulates over time and can initiate catabolic cell behaviour (Lin et al., 2004, Riley, 2004, Riley, 2005). To understand the processes behind tendinopathy, a range of model systems have been developed to simulate tendon overuse, characterise the development of fatigue damage, and investigate how this may relate to the aetiology of tendinopathy (Shepherd and Screen, 2013b). *In vitro* models provide very controlled loading conditions, in which to investigate the mechanics of fatigue damage and the nature of tendon failure. Data from these studies have shown strain to be the primary mechanical parameter governing tendon damage accumulation and injury (Schechtman and Bader, 1997, Wren et al., 2003). They have also highlighted that changes in matrix structure proceed non-linearly, accelerating before rupture (Parent et al., 2011) and that the onset of visual matrix damage precedes statistically significant mechanical weakening of the tendon (Fung et al., 2009, Shepherd et al., 2014). The damage hypothesis introduced by Wang is based upon the understanding that damaged material no longer contributes to stiffness or strength whereas intact material makes a full contribution to both (Wang and Ker, 1995).

Whilst *in vitro* tendon fatigue analysis has traditionally considered whole tendon mechanics, a recent body of work has focused on isolated fascicle fatigue (Legerlotz et al., 2013, Maeda et al., 2007, Screen, 2003, Screen et al., 2003, Screen et al., 2005a, Thorpe et al., 2013a, Thorpe et al., 2013b). The fascicle size scale is of benefit, as the testing of viable tendon sections is simpler, enabling investigation into factors such as cellular mechanotransduction responses (Banes et al., 1999a, Banes et al., 1999b) and the role of inflammation (Devkota et al., 2007, Flick et al., 2006). Fascicles can be removed from bulk tendon with relative ease, providing a complete unit with a comparatively consistent cross sectional area for analysis (Shepherd and Screen, 2013b, Thorpe et al., 2013a), in which the considerable issues associated with gripping whole tendon samples can be overcomed. Fascicle testing also allows for far more straightforward imaging of matrix damage generation (Shepherd et al., 2014), and analysis of fatigue effects on tissue micro-mechanics and cellular morphology (Cheng and Screen, 2007, Screen et al., 2004a, Screen et al., 2003, Thorpe et al., 2013a). Considering the extent of variability in biological tissues (Ker, 2007), investigating fascicle characteristics can also ensure inter-animal variation is taken into account.

Previous studies of fascicle micro-mechanics have shown crimp straightening and fibre extension to be the dominant extension mechanisms at low applied strains, with fibre sliding dominating beyond the toe region (Cheng and Screen, 2007, Goulam Houssen et al., 2011, Gupta et al., 2010, Screen et al., 2004a, Thorpe et al., 2013a). In studies across a range of tendon types, including rat tail tendon fascicles, (Cheng and Screen, 2007, Screen, 2008, Screen et al., 2004a, Screen et al., 2003, Screen et al., 2004b) more highly loaded bovine tendons (Screen et al., 2013), and also energy storing and positional equine tendons (Thorpe et al., 2013a, Thorpe et al., 2014a, Thorpe et al., 2014b), local strains along fibres have consistently been reported to be smaller than applied strains, as a result of the composite structure of tendon and reliance on fibre sliding for tendon extension.

However, despite this growing body of data concerning tendon micromechanics, there are relatively few studies directly comparing micromechanics in functionally distinct tendons (Thorpe et al., 2013a, Thorpe et al., 2014a, Thorpe et al., 2014b), with none in the bovine model, and few studies investigating the effects of fatigue damage on the micromechanics of tendon at the fascicle and fibre levels (Thorpe et al., 2014a, Thorpe et al., 2014b). Such comparisons are important, in light of the growing body of evidence outlining structural and mechanical differences between tendons with different mechanical functions (Smith et al., 2002, Stanley et al., 2006, Thorpe et al., 2013a, Thorpe et al., 2012). Whilst data indicates that energy storing tendons are more fatigue resistant, there is still evidence that tendinopathy may arise from mechanical fatigue damage, and there is a need to understand how fatigue damage initiates and propagates in different tendon types, to establish why some tendons are more prone to injury.

In previous micromechanical studies, tenocyte nuclei have been stained with a fluorescent dye and their movement tracked during straining under a confocal microscope. The tenocytes align along the tendon fibres and an assumption is made that the cell movement is the same as that of the fibres. The cell nuclei no doubt provide convenient, regular shaped markers for the analysis of local strains, but the strains recorded will be highly dependent upon the association between the cells and the surrounding matrix (Screen et al., 2004a). Recent studies have considered an alternative approach to characterising tissue micro-strains, in which the collagen matrix is stained, a grid photo-bleached onto the collagen, and the deformation of the grid in response to applied strain monitored (Cheng and Screen, 2007, Thorpe et al., 2013a, Thorpe et al., 2014a). However, it currently remains unclear how comparable measures taken with these two techniques may be. This work therefore had three key aims:

1. To investigate micro-scale extension mechanisms in fascicles from two functionally distinct tendons of the bovine hoof – the deep digital flexor and the digital extensor.
2. To investigate the effects of fatigue loading on fascicle micro-mechanics, hypothesising that the relative importance of fibre extension and fibre sliding within a fascicle will change as a result of fatigue damage. It is also hypothesised that fatigue damage, and thus changes in micro-mechanics, will be less significant in the more energy storing flexor tendon.
3. To compare two techniques for characterising micromechanics: bleaching a grid on the matrix to directly measure collagen deformation, and using the cells as fiducial markers of fibre movement.

## 2 Materials and methods

### 2.1 Tendon source and fascicle dissection

The feet of healthy bovines (male steers between 18 and 36 months of age) with no observed tendon injury were sourced from a local abattoir. The tensional regions of the three digital extensor tendons and the deep digital flexor tendon were removed within 24 h of slaughter. Tendons were either frozen immediately upon dissection (−20 °C; max duration of 30 days) or used within 12 h of removal.

Upon defrosting and during fascicle dissection, hydration was maintained with Dulbecco׳s Modified Eagles Medium (DMEM). Fascicles of length at least 20 mm were carefully dissected and maintained under DMEM hydration until use. Previous studies have shown no difference in the mechanical properties of fascicles from any of the three bovine extensor tendons in the hoof so these were pooled (Screen, 2003). Further, short-term freezing (less than 30 days) has been shown to have minimal effect on tendon mechanics (CHOU and NG, 2003, Giannini et al., 2008, Ng et al., 2005), so fresh and frozen samples were pooled for analysis. Fascicles from the central regions of all tendons were selected only, as a variation in the mechanical properties of fascicles from different regions of the human patellar tendon has previously been demonstrated (Haraldsson et al., 2005). Fascicle diameters were measured along a 10 mm length in the centre of each fascicle using a laser micrometre with a resolution of 0.5 μm (Mitutoyo, Kawasaki, Japan). The minimum value of approximately 25 measurements over this 10 mm length was used to calculate the cross-sectional area assuming a circular cross-section. This is a method commonly applied by the authors and the assumption of circular cross-section has been shown previously to result in a relatively small overestimation in cross-sectional area of no more than 4% (Thorpe et al., 2012).

### 2.2 Fatigue loading

Individual custom designed stainless steel loading chambers (grip-to-grip distance 10 mm) were used for cyclic creep loading; these allowed the fully hydrated testing of single tendon fascicles without slippage (Legerlotz et al., 2013). Each chamber was filled with DMEM and the chambers were secured in a mechanical loading frame (either a BOSE ElectroForce (BOSE Corporation, Eden Prairie, MN, USA) or an Instron Electropuls dynamic loading system (Instron, Norwood, MA)). Significant prior investigation confirmed that for tests of duration less than 90 min, consistent results are achieved with the use of either machine even with the difference in testing temperature (37 °C in the case of the Bose and room temperature for the Instron).

Fascicles were loaded to a peak stress of 25% of the mean UTS (28.8 MPa in the case of the extensor and 19.7 MPa in the case of the flexor). Loading was carried out with a sine wave form at a frequency of 1 Hz, monitoring displacement throughout the test. This loading protocol has been shown previously by the authors to generate fatigue damage after short testing periods (Shepherd et al., 2014). Tests were stopped at 300 or 900 cycles, after which fascicles were either subjected to a quasi-static test to failure, or prepared for confocal imaging and micro-strain analysis. Quasi-static tests were carried out without removal of the fascicles from the chambers, using the Instron Electropuls system. Tests were carried out at a 1 mm/s extension rate with a 250 N load cell.

### 2.3 Confocal imaging and micro-strain analysis

Confocal imaging and micro-strain analysis were carried out using a custom designed rig developed by Screen et al. (2003) allowing the controlled strain of fascicles, whilst visualising the response on a confocal microscope. All fascicles were strained in 2% increments to a maximum strain of 10% at a rate of 1%/s, following a straining method described by Thorpe et al. (2013a).

### 2.4 Tenocyte nuclei as fiducial markers

After 0, 300 or 900 cycles of creep loading, fascicles were incubated in 5 μM Acridine Orange (Invitrogen, Eugene, Oregon, USA) in DMEM for 40 min, to stain the cell nuclei. After repeated washing in phosphate buffered saline (PBS) to ensure removal of excess stain, fascicles were then loaded into the custom designed rig. Fascicles were secured at an initial test length of 10 mm, and the grips moved out until the fascicle straightened and was observed (through the microscope eye piece) to lift off the base of the rig (Screen et al., 2003). This point was defined as zero strain. Imaging was carried out at room temperature on an inverted Perkin Elmer Ultraview Spinning Disc Confocal system with a Nikon Eclipse TE300 Microscope.

**Fig. 1:**
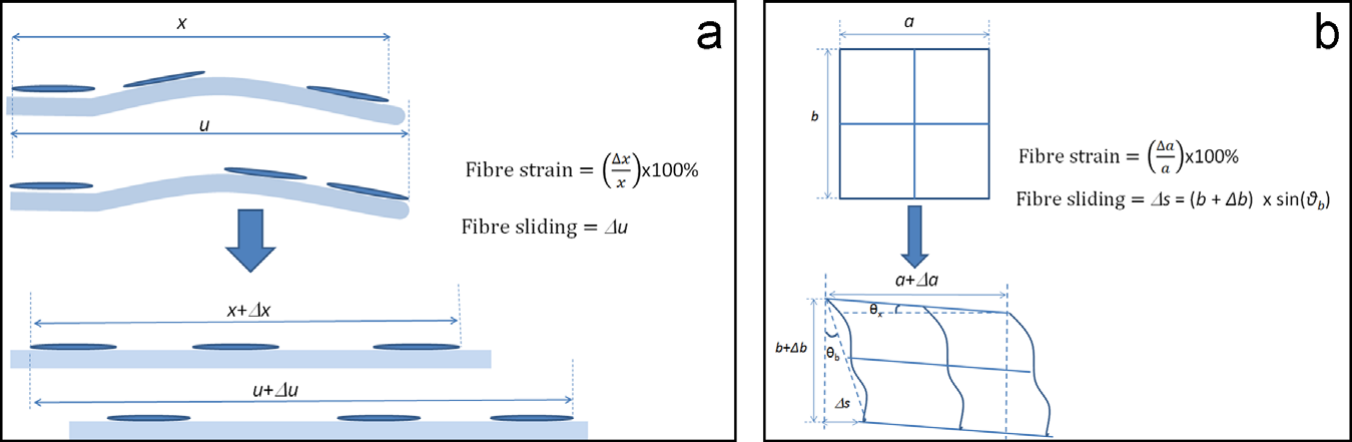
Methods of measuring fibre strain and fibre sliding using: (a) nuclei as fiducial markers; and (b) a photo-bleached grid.

Following previously reported protocols (Rasband, 1997–2011, Screen et al., 2004a, Screen et al., 2003), rows of tenocytes were selected in a single focal plane approximately 50 μm below the surface of the fascicle, and imaged during straining, taking a photo of the same cell groups at each increasing strain increment. At least two rows of tenocytes were considered in each fascicle to allow investigation of both fibre strain and fibre sliding (Fig. 1a). Imaging was carried out with a 20× objective aperture and nuclei were selected with a separation as large as possible so as to minimise errors during measurement. Imaging was carried out 1 min after incremental load was applied to allow for relaxation of the sample. Measurement of nuclei separation was carried out using ImageJ (Rasband, 1997–2011) with Δ*u* (fibre sliding) measured directly and fibre strain calculated using Eq. (1) (see Fig. 1)

(1) $$\mathrm{Fibre}\;\mathrm{strain}=\;\left(\frac{\Delta x}{x}\right)\times 100\%$$

### 2.5 Deformation of photo-bleached grid

After 0, 300 or 900 cycles of creep loading, additional fascicles were stained with a 2 mg/ml solution of 5-(4,6-Dichlorotriazinyl) Aminofluorescein (5-DTAF) in 0.1 M sodium bicarbonate buffer at pH 9 for 20 min in order to stain the collagen. After repeated washing in PBS, fascicles were loaded into the custom designed rig described above, and a grid photo bleached onto the sample, as previously described using a Leica SP5 confocal microscope (Thorpe et al., 2013a). Briefly, a grid consisting of four 50 μm×50 μm squares was photo-bleached, with 2 μm thick lines, using a 488 nm krypton-argon laser and the 20× objective aperture. The laser power was reduced and imaging also carried out using the same objective aperture. An image of the photo-bleached grid was taken approximately 50 μm below the surface of the fascicle, after which the fascicle was strained in 2% increments, imaging the grid at each increment. Image analysis was carried out based on the previous work of Thorpe et al. (2013a) but in order to correlate with the nuclei deformation data only grid extension parallel to the strain direction (fibre strain – Eq. (2) and deviation of the horizontal grid lines (fibre sliding – Eq. (3) was considered, as demonstrated in Fig. 1(b).

(2) $$Fibre\;strain=\;\left(\frac{\Delta a}{a}\right)\times 100\%$$

(3) $$Fibre\;sliding=\Delta s=\;(b+\Delta b)\times \;\sin(\theta_{b})$$

### 2.6 Statistics

Normality of data was investigated using the Shapiro–Wilk test. Statistical significance was then determined appropriately using a student *T*-test or single factor analysis of variance (ANOVA) followed by Tukey׳s post-hoc test for normally distributed data sets. A Kruskal–Wallis test followed by a Mann Whitney test (IBM SPSS software) was used for other data sets.

For each of the three post-creep tests (quasi-static test to failure and micro-strain analysis with either nuclei as fiducial markers or a photo-bleached grid) at least 10 fascicles from at least 3 independent tendons were tested from each tendon type (extensor and flexor) after 0, 300 and 900 cycles. At least 30 fascicles from each tendon type where therefore considered in total. Approximately 50% of the fascicles considered were used after freezing, evenly split between the two micro-scale mechanics analysis methods and the macroscopic mechanical properties. No statistically significant differences between frozen and fresh samples were seen for any of the test groups, so these are pooled in the results. Statistical significance was taken as *p*<0.05.

## 3 Results

### 3.1 Macroscopic mechanical properties

**Fig. 2:**
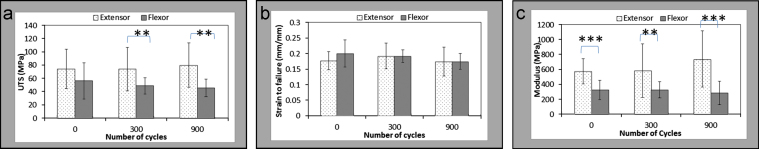
Macroscopic mechanical properties of bovine digital extensor and flexor tendon fascicles: (a) ultimate tensile strength (UTS); (b) strain to failure (mm/mm); (c) Tensile modulus (MPa). Statistical significance was carried out with Student׳s *T* test: ^⁎⁎^*p*≤0.01, ^⁎⁎⁎^*p*≤0.001. There is statistically significant variation in both the UTS and tensile modulus of flexor and extensor tendon fascicles prior to fatigue loading. However, no statistically significant variations in mechanical properties of either fascicle type are observed with fatigue loading.

**Fig. 3:**
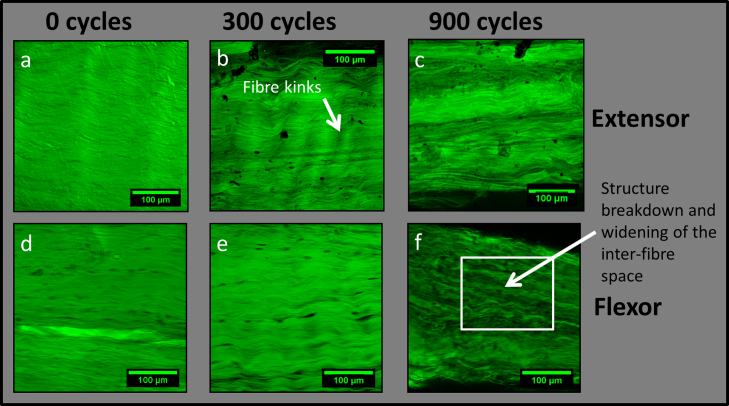
Confocal images of extensor (a–c) and flexor (d and e) fascicles after creep loading at 25% UTS through 0, 300 and 900 cycles. Fibre kinking is observed at 300 cycles, whilst variation in staniing intensity indicatewidening of interfibre spacing and structure breakdown after 900 cycles in both fascicle types.

Data from the quasi-static tests to failure are detailed in Fig. 2. Whilst significant differences were evident in both the ultimate tensile strength and the tensile modulus (maximum gradient of the stress–strain curve) of the two different tendon types, cyclic creep loading had no significant effect on any of the macroscopic mechanical properties of the fascicles. This was despite the observation of reproducible and extensive matrix damage, such as that shown in Fig. 3*,* particularly in the case of the extensor tendon fascicles. Fibre kinking, a characteristic early damage indicator was observed after just 300 cycles of creep loading in the extensor tendon fascicles, with widening of inter-fibre space and more significant matrix structure breakdown after 900 cycles. Localised matrix structure breakdown was also clearly observed after 900 cycles of creep loading in the case of the more energy storing flexor tendon.

### 3.2 Micro-strain analysis

**Fig. 4:**
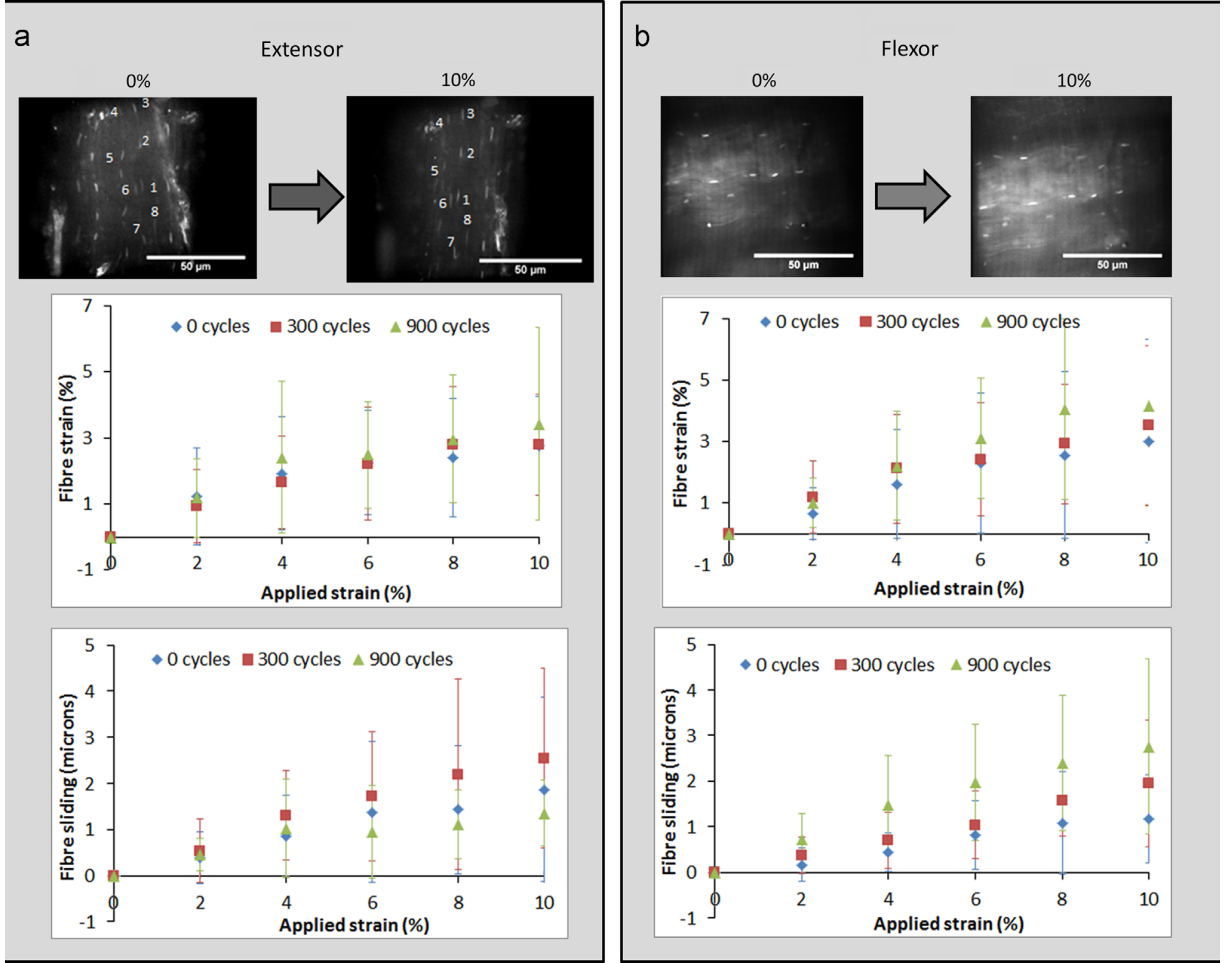
Micro-deformation as measured using the tenocyte nuclei as fiducial markers: (a) shows the deformation within extensor fascicles (images at 0% and 10% applied strain) and (b) within flexor fascicles (again with images at 0% and 10% applied strain). The nuclei used for analysis in this particular extensor sample are numbered. Neither fibre strain nor sliding was significantly affected by fatigue damage caused by creep loading.

As observed previously (Screen et al., 2003), bovine tendon fascicles suffer from significant autofluorescence, so contrast between the nuclei and matrix in acridine orange stained fascicles is less pronounced than observed in the rat tail tendon. However, in all samples it was possible to identify sufficient nuclei in a single plane for micro-deformation analysis using the nuclei tracking technique. The number of nuclei appeared broadly independent of fatigue loading, although in regions of severe damage it was not possible to identify nuclei on the same or adjacent fibres and thus analysis could not be carried out in these regions. Fig. 4 summarises micro-mechanics data from digital extensor fascicles (Fig. 4a) and digital flexor fascicles (Fig. 4b), monitoring cell nuclei displacement under increasing applied strains. Fibre strains were the same for both extensor and flexor fascicles, and surprisingly were not affected by fatigue loading in either tendon type. In all cases, fibre strains increased rapidly with the initial application of strain, levelling off as further strain was applied, to reach a maximum mean value of approximately 4% fibre strain at 10% applied strain. No differences in fibre sliding were observed between extensor or flexor fascicles either with or without the application of fatigue loading. Measured fibre sliding was generally small, with a maximum mean displacement of only 3 μm in the case of the flexor fascicle after 900 cycles creep loading.

**Fig. 5:**
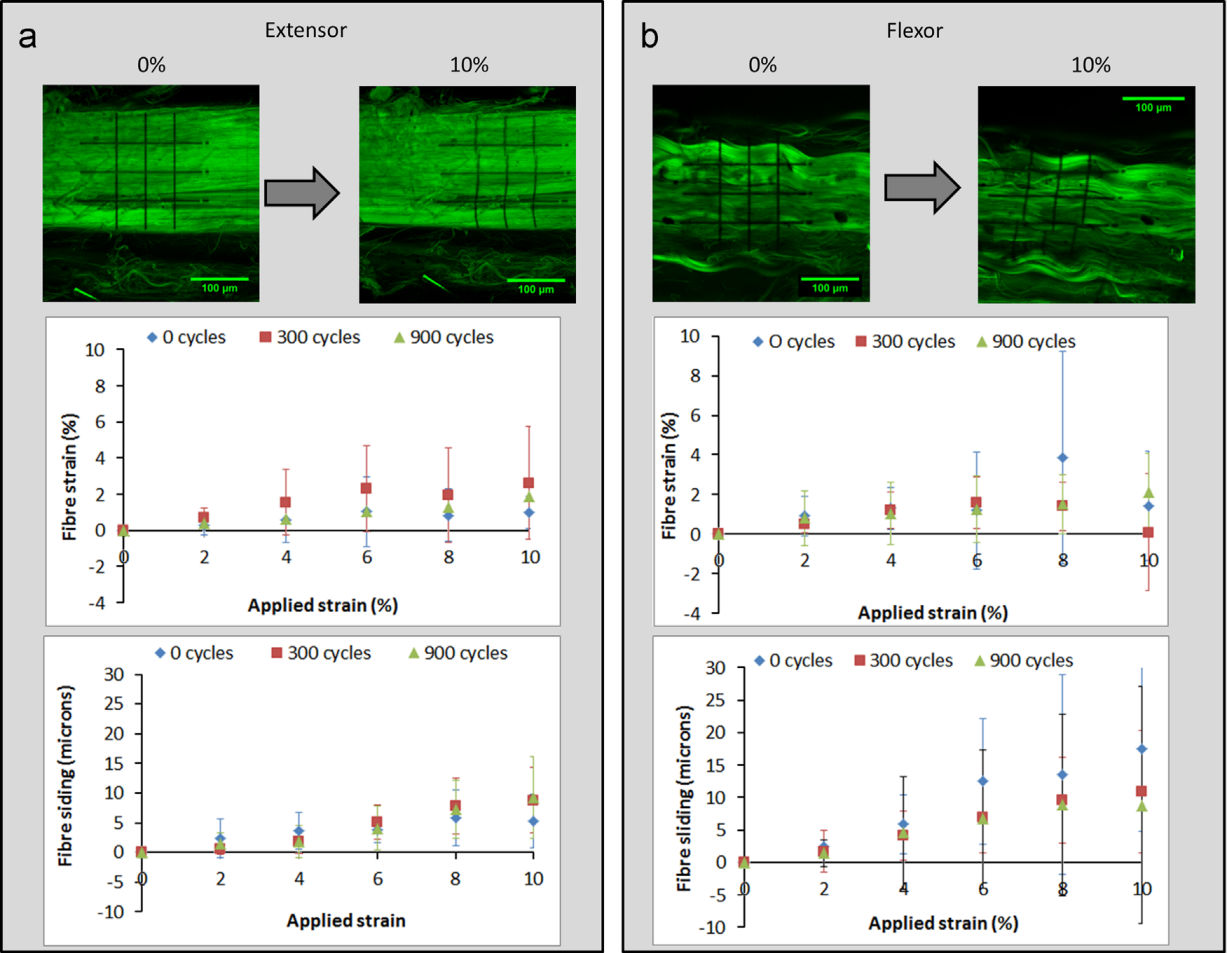
Micro-deformation as measured using the deformation of a photo-bleached grid: (a) shows the deformation within extensor fascicles (images at 0% and 10% applied strain) and (b) within flexor fascicles (again with images at 0% and 10% applied strain). Neither fibre strain nor sliding was significantly affected by fatigue damage caused by creep loading.

Fig. 5 summarises the equivalent micro-mechanics data as measured from deformation of the photo-bleached grid. Once again, no significant differences in extension mechanisms between tendon types were apparent, with or without fatigue loading. However, whilst not a focus of this work, it was noted that after fatigue loading, perpendicular strains were higher in extensor than flexor fascicles (*p*<0.05).

**Fig. 6:**
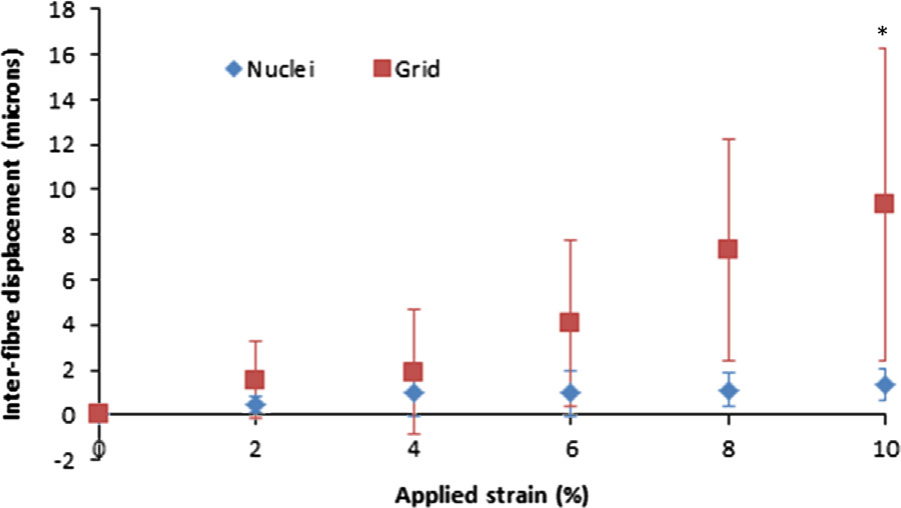
Comparison of the two methods for determination of fibre slding after 900 cycles of creep loading for the bovine digital extensor tendon. Data highlights that fibre sliding measures were consistently higher when measured using a grid, rather than cell nuclei displacements. Statistical significance was observed between the two testing routes at 10% strain (^*^p<0.05). Statistical significance was also observed between the two testing routes after 300 cycles of creep loading for both extensor and flexor tendons at 6%, 8% and 10% applied strain.

Comparing the two methods for measuring micro-mechanics, fibre strain as measured using the two routes was broadly consistent. However, larger magnitudes of fibre sliding were measured with the grid deformation route than using the nuclei as fiducial markers (Fig. 6). Statistical significance was observed between the two testing routes at 8% strain for bovine digital extensor tendons after 900 cycles of creep loading. Statistical significance was also observed between the two testing routes after 300 cycles of creep loading for both extensor and flexor tendons at 6%, 8% and 10% applied strain.

## 4 Discussion

This work utilised relatively well developed techniques of micro-strain analysis to investigate the effects of fatigue loading on fascicles from two functionally distinct bovine tendons: the digital extensor and deep digital flexor. It is the first time that micro-strain has been investigated after fatigue for these two tendon types and indeed the first time, micro-scale mechanics have been investigated to any degree in the bovine digital flexor tendon. The work has also carried out the first comparison of two different routes for characterising micromechanics: bleaching a grid on the matrix to directly measure collagen deformation and using nuclei as fiducial markers of fibre movement.

Previous work from the authors (Shepherd et al., 2013a) has compared the fatigue behaviour of fascicles from the bovine digital flexor and extensor tendon, attempting to correlate mechanical changes with structural ones. This previous study indicated a tendency towards improved cyclic creep resistance in bovine flexor tendon fascicles. However, despite the improved creep resistance of the flexor tendon, the current data highlights that mechanical testing after cyclic creep showed no significant differences in the mechanical properties of either the flexor or extensor fascicles. By contrast, the quasi-static mechanical properties of flexor and extensor fascicles were significantly different, before and after fatigue loading. Previous work in the equine model has similarly shown a lower UTS and modulus in flexor tendon fascicles at both the whole tendon and fascicle level; a likely response to the need for greater extensibility in energy storing tendons (Thorpe et al., 2012).

Data in the current study concurs with previous work, showing that visual matrix damage precedes a reduction in the macroscopic mechanical behaviour of the fascicles (Fung et al., 2010, Fung et al., 2009). However, we had hypothesised that micro-scale deformation routes would have less tolerance to matrix breakdown, and would be altered by fatigue loading. No significant changes in sample micro-mechanics were observed with increasing numbers of creep cycles as measured with either technique, despite the matrix damage observed in creep-loaded samples, particularly after 900 cycles (Fig. 3). The large error bars present across the micro-strain data clearly highlight the inhomogenous strain response observed throughout the matrix, and consistent with previous studies, large variability was observed between samples (Cheng and Screen, 2007). The apparent absence of any effect of creep on the measured microstructural deformation may relate to both this variability and the localised nature of the measurement techniques. At the sites where the matrix structure broke down, tracking of the nuclei or photo-bleaching the grid proved problematic, and it is these regions where the greatest degree of deformation would perhaps be expected, with remaining areas of tissue protected from significant levels of stress. Indeed, it should also be recognised that micro-structural extension mechanisms were analysed under displacement control conditions and without measurement of the loads associated with these displacements. Whilst this is the method most commonly applied, perhaps if the tests had been run under load control, a difference would have been more apparent. However, such testing is complex, and careful control of imaging time necessary, to ensure the same time point in the creep curve is continually viewed.

It is also of course necessary to recognise the sources of variability within the study including inter-animal variation, the effect of freezing, testing temperature, cross-sectional measurement and the definition of zero strain. Analysis of the data highlighted no significant inter-animal differences, and whilst a number of studies investigating the effect of freezing on tendon mechanics have shown both moderate increases (Ng et al., 2005) and decreases (Clavert et al., 2001, Giannini et al., 2008) in the ultimate tensile strength, no significant differences were recorded between fresh and frozen samples in the current study. This finding may relate to the time period over which fascicle were frozen; previous studies have generally considered the effects of long term sample freezing (well over 30 days), whilst all samples in the current study were frozen for shorter time periods.

Fatigue loading was carried out using two machines at different temperatures, the Instron at room temperature and the Bose system in an incubator at 37 °C. Fatigue resistance is known to be affected by testing temperature (Wang and Ker, 1995), however, in the current study, testing was carried out over a short time period, and no effects of temperature on either samples fatigue properties or cell viability were evident.

The large variations observed in both macro- and micro-scale fascicle mechanics may in part be a result of the use of cyclic creep to fatigue samples prior to analysis. The UTS of tendon fascicles has been shown to vary significantly, meaning the stress selected for cyclically loading samples in the current study will actually encompass a range of percentages of UTS across the individual fascicles. Indeed, in a recent study that attempted to predict the elastic strain limit of tendons, Reyes et al observed that out of 97 explants tested to failure, the UTS of 4 was below 2 standard deviations from the mean, while no such outliers were found when looking at failure strain (Reyes et al., 2014).

Whilst fatigue loading under stress relaxation conditions is therefore likely to offer more reproducible data, *in vivo* loading conditions are complex and loading is unlikely to be a purely displacement controlled phenomenon. As such, it is important not to neglect damage generation through creep loading.

During creep, fibre bundles are recruited and stretched out progressively (Abrahams, 1967, Sereysky et al., 2012, Thornton et al., 1997). Previous studies have shown that damage appears to be initiated in the non-collagenous matrix (Neviaser et al., 2012, Shepherd et al., 2013a, Shepherd et al., 2014, Thorpe et al., 2014a) with fibre damage only reported at very high levels of fatigue (Neviaser et al., 2012, Shepherd et al., 2014). It seems likely that such damage would be evident in the analysis of fibre sliding in fatigued samples. However, studies in the equine model have suggested that other extension mechanisms may come into play in tendon fascicles. A previous study by Thorpe et al. (2014a) considered fascicle micromechanics post-fatigue loading in the highly energy storing superficial digital flexor tendon in young and old horses, and showed that neither fibre sliding nor fibre extension were altered by fatigue loading in young horses, in direct agreement with the data shown here. However, their study additionally considered fascicle rotation, and reported that this micro-scale deformation mechanism was affected by fatigue loading in tendons from young horses. In contrast, a significant increase in fibre sliding was observed with fatigue in old horses. It was hypothesised from these data that fascicles from young energy storing tendons have a helical structure to help improve their resilience to fatigue loading, and it was only after this helical structure was damaged (in old horses) that matrix damage (increased fibre sliding) began to predominate. The tendons in the current study were from the feet of healthy male steers between 18 and 36 months of age, so whilst bovine flexor tendons do not undertake an energy storage role as extreme as that of horse flexor tendons, tendon microstructure may have some similarities with that of the young horse. As such, rotation of the fascicles should perhaps be considered in future studies, as it is possible that a similar helical structure prevented the immediate accumulation of microstructural damage in these young bovine fascicles.

Considering the grid deformation measure of micro-strains, beyond the toe region, fibre sliding dominated tendon extension, both with and without creep loading. This same move towards dominant fibre sliding was less evident with nuclei tracking techniques, with this method consistently reporting lower levels of fibre-sliding than measured from grid deformation, at applied strains greater than 6%. Through use of nuclei as fiducial markers, an assumption is made that cells remain attached to the collagen fibres throughout loading. Whilst there is no doubt that mechanical loading is perceived by the resident cells and this has been shown to elicit a cellular response (Franchi et al., 2007, Kjær et al., 2009, Wang, 2006), it is not known how well the tenocytes adhere to the collagen fibres. The current data indicates that there may be a passive relative displacement between the matrix and the cells. Cell viability was not investigated during this work and further investigation is required in order to establish whether the indirect relationship between collagen fibre and cell movement is a form of protection mechanism for the cell, or whether cell viability is compromised, and the interaction between cell and fibre is broadly lost. Arnoczky et al have suggested that an alteration of cell–matrix interaction as a result of fibrillar damage could result in a reduced mechanical stimulation of the resident tendon cells which could actually initiate further degeneration of the matrix (Arnoczky et al., 2007, Lavagnino et al., 2003). Understanding the interactions between cell and matrix are therefore of prime importance.

Previous use of nuclei as fiducial markers has shown contradictions with this current data, with fibre sliding becoming more significant at higher applied strains and reaching up to 3.9% of the applied strain (Screen et al., 2004a). However, these studies have utilised rat tail tendon fascicles (Screen, 2008, Screen et al., 2004b, Screen et al., 2005b) where fascicle removal occurs with ease and is likely to place considerably less stress on the tissues and resident cells during dissection. Further, our data across a number of studies has highlighted the differences in mechanics across functionally distinct tendons, making it difficult to directly compare these studies.

## 5 Conclusions

Fatigue loading of fascicles from both the bovine digital flexor and extensor tendon generated visual matrix damage after as little as 300 cycles. However, these visual changes did not appear to affect either gross fascicle mechanics or fascicle microstructural extension mechanisms. The apparent absence of any affect of creep on the measured microstructural deformation may be a result of the localised nature of the measurement techniques and the use of displacement controlled loading during analysis. Alternatively, data may confirm that fascicles can withstand a degree of matrix disruption with no impact on mechanics. In terms of measuring marix deformation, photobleaching a grid to directly monitor collagen is apparently the superior route. However, the strain felt directly by the cells is undoubtedly of importance, and nuclei tracking provides a useful method of monitoring this.
